# Feeding a *Saccharomyces cerevisiae* Fermentation Product (Olimond BB) Does Not Alter the Fecal Microbiota of Thoroughbred Racehorses

**DOI:** 10.3390/ani12121496

**Published:** 2022-06-08

**Authors:** Alexandra Lucassen, Julia Hankel, Christa Finkler-Schade, Lisa Osbelt, Till Strowig, Christian Visscher, Hans-Joachim Schuberth

**Affiliations:** 1 Institute of Immunology, University of Veterinary Medicine Foundation, 30559 Hannover, Germany; alexandra.lucassen@tiho-hannover.de; 2 Institute of Animal Nutrition, University of Veterinary Medicine Foundation, 30559 Hannover, Germany; julia.hankel@tiho-hannover.de (J.H.); christian.visscher@tiho-hannover.de (C.V.); 3 Schade & Partner, 27283 Verden, Germany; cs@schadeundpartner.de; 4 Helmholtz Center for Infection Research, Inhoffenstraße 7, 38124 Braunschweig, Germany; lisa.osbelt@helmholtz-hzi.de (L.O.); till.strowig@helmholtz-hzi.de (T.S.); 5 Hannover Medical School, Carl-Neuberg-Straße 1, 30625 Hannover, Germany

**Keywords:** *Saccharomyces cerevisiae*, prebiotics, postbiotics, microbiome, neutrophilic granulocytes, vaccination, horses

## Abstract

**Simple Summary:**

*Saccharomyces cerevisiae* fermentation products (SCFP) are feed supplements and are widely used in animal nutrition to promote health. The biological effects of SCFP are based on prebiotic mechanisms that directly influence the microbial community of the gut microbiome or postbiotic factors that directly interact with host cells. To show whether the immunomodulatory effects of SCFP feeding are due to an altered composition of gut microbiota, we analyzed the fecal microbiota of racehorses. Horses were fed either the SCFP (Olimond BB) or a placebo product for six weeks, and fecal samples were collected for 16S rRNA gene sequencing. During this period, SCFP feeding only subtly affected the fecal microbiota in bacterial composition and diversity. SCFP and placebo horses differed significantly in the fecal bacterial diversity directly after intramuscular influenza vaccination. Altogether, the findings argue against a strong prebiotic effect of SCFP in racehorses. In contrast, the modulation of vaccine- and host-induced alterations of the microbiome suggests that the main effects of SCFP are due to contained or induced postbiotic components.

**Abstract:**

Feed supplements such as *Saccharomyces cerevisiae* fermentation products (SCFP) alter immune responses in horses. The purpose of this study was to analyze whether a prebiotic activity of the SCFP alters the gut microbiome in horses. Racehorses were fed either SCFP (Olimond BB, OLI, *n* = 6) or placebo pellets (PLA, *n* = 5) for 43 days. Fecal microbiota analysis was performed using 16S rRNA gene sequencing. The numbers and function of circulating immune cell subpopulations were analyzed by flow cytometry. SCFP supplementation resulted in non-consistent differences in fecal microbiota between the PLA and OLI during the feeding period. Rather, the individual animal had the highest impact on fecal microbiota composition. OLI and PLA horses displayed the same changes in numbers of blood leukocyte subpopulations over time. One day after a booster vaccination against equine influenza during the feeding period, the alpha diversity of fecal microbiota of PLA horses was significantly higher compared to OLI horses. This suggests that SCFP feeding altered the vaccination-induced spectrum of released mediators, potentially affecting gut microbiota. The overall non-consistent findings argue against a strong prebiotic effect of Olimond BB on the microbiota in racehorses. Fecal microbiota differences between the groups were also noticed outside the feeding period and, hence, are most likely not caused by the SCFP additive.

## 1. Introduction

In symbiosis with the host, gut microbiota provide energy and other essential nutrients by metabolizing host-indigestible cellulose and hemicellulose feed components [1]. Microorganisms interact with resident immune cells, intestinal epithelial cells, and neurons of the enteric nervous system [2] and release immunomodulatory molecules affecting the functional polarization of the resident immune cells [3]. Metabolic products of the germs (postbiotics) are also resorbed and distributed in the body and modulate various immune mechanisms and cell differentiation processes in the periphery [4]. Changes in the intestinal microbiome can lead to altered immune mechanisms in various parts of the body [2]. The composition of the gut microbiome can be altered via feed or by supplementing the feed with distinct additives [2]. One class of feed supplements is based on fermentation products of *Saccharomyces cerevisiae* (SCFP, *Saccharomyces cerevisiae* fermentation products). SCFPs were reported to have effects on the microbiota of the digestive tract with positive consequences for the health of the organism. In lactating dairy cows, SCFP feeding promoted the relative abundances of certain bacterial communities of the rumen milieu and attenuated the negative effects of subacute rumen acidosis [5]. In calves, SCFP feeding altered the microbial species richness in the rumen and colon [6] and favored the gut development and morphology [7]. Such prebiotic effects with an expansion of commensal bacteria and a reduced abundance of pathogenic bacteria were also seen in SCFP-fed dogs [8]. Adult cats showed a prebiotic effect by reducing *Clostridium perfringens* in the fecal microbiota [9]. So far, the effect of SCFP feeding on the equine gut or fecal microbiota has not been investigated.

Data for feed-induced changes in the equine microbiome are available for highly concentrated grain feed [10,11], high-starch feed [11], haylage [12], and grass-based diets [10]. Whether food supplements induce changes in the equine gut microbiome is less well studied. Feeding multi-strain probiotics to foals caused limited changes in the relative abundance of bacterial families, with the enrichment of *Lactobacillus* in the probiotic group [13]. In adult horses, a combination supplement of yeasts (*Saccharomyces cerevisiae*) and microalgae (*Aurantiochytrium limacinum*) increased the activity of fibrolytic bacteria, which counteracted the dysbiosis caused by high starch intake [14]. Other studies reporting on the questionable or lacking evidence of positive clinical effects of probiotic feed supplements in horses did not analyze gut or fecal microbiota-altering effects [15,16].

In horses, SCFP feeding exerted immunomodulatory effects. Ponies fed for 8 weeks with an SCFP product displayed a more rapid decrease in cortisol levels after exercise and little or no exercise-increased serum amyloid A or cytokine levels [17]. The SCFP feeding of young horses for 84 days resulted in lower prostaglandin E2 levels in the synovium after LPS injection into the carpal joint [18]. Previously, we reported that feeding SCFP to racehorses altered the circulation behavior of blood leukocyte subpopulations after an influenza booster [19]. Functional interactions between the gut microbiome and the vaccination response were first described in humans and mice [20,21,22,23]. In piglets, the composition of the microbiota before vaccination was predictive of the quantitative response after influenza A virus vaccination [24]. In bovines, a significant association was observed between bacterial communities of the bovine fecal microbiota and the immune response after parenteral vaccination against *E. coli* O157:H7 [25].

Whether the SCFP-induced changed immune response towards vaccination in racehorses was due to a gut microbiome-altering prebiotic effect or whether it was dominated by postbiotic SCFP components or SCFP-induced postbiotic products is still unclear.

The main aim of this study was to analyze putative SCFP feed-induced changes in the gut microbiome based on the characterization of the fecal microbiota composition over a ten-week trial with a six-week period of SCFP supplementation. Further, we analyzed whether SCFP feeding alters the leukocyte homeostasis in blood and a putative vaccine challenge-induced alteration of the fecal microbiota.

## 2. Materials and Methods

### 2.1. Animals

Eleven horses of the English Thoroughbred breed participated in the trial, which was conducted at a stud farm in North Rhine-Westphalia, Germany. The trial was approved by the State of North Rhine-Westphalia (Ref: 81-02.04.2020.A177) in accordance with §8 (1) of the Animal Welfare Act in conjunction with §33 of the Animal Welfare Experimental Animal Ordinance. The owner of the horses agreed to their participation in the form of a signed declaration prior to the study. The horses (10 mares and 1 stallion aged two to three years) had a mean body weight of 442 kg ± 34 kg. The horses were kept in individual stalls (3.50 m × 3.50 m) and received ad libitum grass hay and water access as well as oats (2.7 kg average daily amount), a concentrate (0.9 kg average daily amount), and minerals (0.2 kg daily amount), in three meals/day for moderate exercise. The grain-based concentrate contained 12.8 MJ DE, 20% crude protein, 8.5% crude fiber, and 7% oils. The horses were exercised every day. The training was performed at different intensities: walking in a horse walker, trotting on the racetrack, slow canter on the racetrack, brisk canter on the racetrack, or simulation of a race on the racetrack. All of the horses underwent the same training at different frequencies so that, on average, they were trained for the same length of time and with the same mean intensity throughout the study.

### 2.2. Supplementation

**Figure 1:**
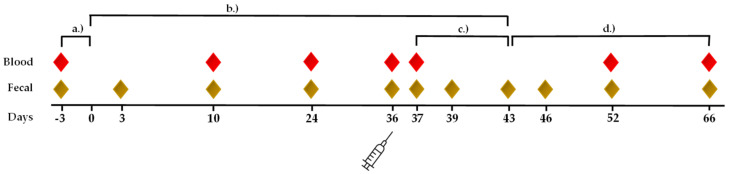
Timeline and sampling days of the trial. Blood samples (red diamond) and fecal samples (brown diamond) were collected on the indicated days. (**a**) day −3 to 0 before supplementation. (**b**) day 1–43, the horses received the SCF product (OLI, *n* = 6) or a placebo (PLA, *n* = 5) during supplementation. (**c**) day 37–43 after vaccination. (**d**) day 43–66, the horses received no supplementation (after supplementation period). On day 36, the horses were vaccinated parenterally against equine influenza.

The animals were randomly divided into two groups. Five horses (PLA group) were fed placebo pellets (10 pellets/day), and six horses (OLI group) were additionally fed Olimond BB pellets (10 pellets/day). The composition of the PLA and OLI pellets (5.01 g each) is given in Table S1. Both pellets contained equal amounts of tocopherol extract, coconut oil, and vitamin C. The content of microcrystalline cellulose (PLA 1.03 g) and minerals (PLA 1.13 g) was slightly higher in OLI pellets (1.43 g, 1.23 g). OLI pellets contained 2 g of inactivated yeast and yeast fermentation products. In PLA pellets, yeast products were replaced by dextrose, corncob meal, and linseed cake (Table S1). The OLI and PLA pellets were of the same size and color. The study was double-blinded. The pellets were given daily at the same time (evening) as the basic diet. The feeding period lasted 43 days (Figure 1).

### 2.3. Blood Samples and Body Temperature

The blood was collected by venipuncture (external jugular vein) in BD Vacutainer^®^ sodium heparin tubes (Becton Dickinson, Heidelberg, Germany) as described [19] (Figure 1). The samples were processed and analyzed within 24 h after collection. Prior to the blood collection, all of the horses were examined for general health. Rectal body temperature was measured daily in the morning with a digital thermometer (Microlife^®^ Vet-Temp Thermometer, Covetrus, Hamburg, Germany).

### 2.4. Blood Leukocyte Preparation and Subpopulation Determination

The recording of the total numbers of blood leucocytes and the flow cytometric determination of dominant subpopulations among leukocytes (lymphoid cells, granulocytes, monocytes) and reticulocytes have been described in detail [1,19]. In brief, after the hypotonic lysis of heparinized blood, nucleated blood cells were identified as lymphoid cells, granulocytes, or monocytes based on their characteristic scatter characteristics after flow cytometric acquisition. Their relative fraction among the leukocytes was multiplied by the total numbers of leukocytes/mL blood to obtain the absolute values for each major subpopulation.

### 2.5. Flow Cytometric Determination of Lymphocyte Subpopulations and Reticulocytes

To determine lymphoid subpopulations, leukocytes were incubated with equine-specific and equine cross-reactive monoclonal antibodies, as described in detail in [19]. In brief, leucocytes obtained after the hypotonic lysis of heparinized blood were incubated in separate setups on ice for 10 min with antibody mixtures: Mixture 1: anti-eqCD4-FITC, anti-caCD21-AlexaFluor^®^ 647, anti-eqMHC-II-RPE. Mixture 2: anti-eqCD4-FITC, anti-eqCD8-RPE. Mixture 3: murine IgG isotype controls IgG1-FITC, IgG2a-PE). All monoclonal antibodies were from BIO-Rad (Fedlkirchen, Germany). After labeling, the washed cells were flow cytometrically analyzed, and after gating on the viable and single cells [19], the fractions of CD4^+^ T cells, CD8^+^ T cells, CD21^+^ B cells, and CD21^−^/MHC-II^+^ lymphoid cells were determined. To obtain total numbers/mL, the fraction of the subpopulation among lymphoid cells was multiplied by the absolute number of lymphoid cells/mL in the blood. The determination of the reticulocyte fractions among erythrocytes was conducted according to (2) as detailed in [19]. In brief, the heparinized blood was mixed with 2 mL of PBS and 20 µL of acridine orange solution (5 µg/mL of PBS) for 30 min in the dark. Reticulocytes among erythrocytes were identified based on their higher fluorescence signal in the FL1 channel.

### 2.6. Vaccination and Determination of Influenza-Specific Antibodies

Five horses from the PLA group and 6 horses of the OLI group were vaccinated intramuscularly on day 36 with an influenza vaccine (PROTEQFLU™, Boehringer-Ingelheim, Ingelheim am Rhein, Germany) containing influenza A/eq/Ohio/03 [H3N8] recombinant canary virus (strain vCP2242) and influenza A/eq/Richmond/1/07 [H3N8] recombinant canary virus (strain vCP3011), with carbomer as adjuvant). All horses were of the same age (2 years) (PLA: 1 stallion, 4 mares; OLI: 6 mares). On the day of vaccination and 24 h after vaccination, the horses were examined for health and side effects (pain, swelling at the injection site). Influenza-specific blood serum antibody titers were determined in an external laboratory (LABOklin GmbH & Co.KG, Bad Kissingen, Germany) in samples taken on the day of vaccination and one day later. The details of the influenza strains used for the ELISA are mentioned in [19].

### 2.7. Fecal Samples for Microbiota Analysis

To determine the fecal microbiota, samples from fresh feces were taken on days −3, 3, 10, and 24 after the start of supplementation, on the day of vaccination (day 36), day 37 (1 day after vaccination), and day 39 (3 days after vaccination). After the end of the supplementation period (day 43), further fecal samples were taken on days 46, 52, and 66 (3, 9, and 23 days after the end of supplementation) (Figure 1). For this purpose, 5 g of feces was taken from the inside of the feces balls with sterile spoons and stored in tubes filled with RNase-Inhibitor (RNAsepar^®^, Biosepar GmbH, Simbach am Inn, Germany) until further analysis.

### 2.8. DNA Extraction

The samples were first purified (ZymoBIOMICS 96 MagBead DNA Kit, Zymo Research Europe GmbH, Freiburg, Germany) before the hypervariable region V4 of the 16S rRNA gene was amplified in accordance with previously described protocols [26]. Therefore, the primer F515/R806 was used. The amplicons were sequenced on the Illumina MiSeq platform (PE300). The Usearch8.1 software package was used to assemble, quality control, and cluster the obtained reads, and -fastq_mergepairs –with fastq_maxdiffs 30 was used to merge the reads. Chimeric sequences were identified and removed with the help of cluster_otus (-otu_radius_pct 3) and the Uchime command included in the Usearch8.1 workflow. Quality filtering was set up with the fastq_filter (-fastq_maxee 1); minimum read length, 200 bp. The reads were clustered into 97% ID operational taxonomic units (OTUs). The OTU clusters and representative sequences were determined with the UPARSE algorithm [27]. Silva database v128 [28] and the RDP Classifier [29] were used for taxonomic assignment with a bootstrap confidence cutoff of 70%.

The samples with fewer than 999 total reads were removed. Chloroplast and Mitochondria as well as OTUs that were not present in at least more than one sample and OTUs with an abundance <0.02% were pruned. After this filtering step, a total of 121 samples were included in the statistical analyses. Six samples stand out because of their high dissimilarity to all other samples. It was noticeable that these samples differed from other samples due to the increased presence of archaea. Therefore, nine taxa belonging to the kingdom archaea were excluded, and only the taxa belonging to the kingdom bacteria were kept. The dataset contained 2,807,473 reads (mean number of reads: 23,202; range: 8569–144,591) mapped to 677 operational taxonomic units (OTUs).

### 2.9. Statistical Analysis

R (version 4.1.2, R Core Team, Vienna, Austria) and the R-package “phyloseq” (version 1.36.0) [30] were used for data visualization and analyses. Selected alpha diversity indices (Observed, Chao 1, and Shannon) were measured with the R-package “phyloseq”. The means of the alpha diversity estimates were compared with the aim of evaluating the influence of the factor supplementation at each time point (Table S2). The data were checked for normality by analyzing the model residuals with the Shapiro–Wilk normality test implemented in the package “rstatix” (version 0.7.0) [31]. The normally distributed data were further tested for equal variance with Levene’s test of the “car” package (version 3.0.12) [32]. Subsequent pairwise comparisons were conducted with the package “rstatix”. Statements of statistical significance were based upon *p*-values < 0.05. The total community structure and composition of samples were assessed during the administration of the supplements (3 days after the introduction of the supplement until 7 days after vaccination = discontinuation of the supplement) for changes in relation to supplementation and animal by permutational multivariate analysis of variance using the Bray–Curtis distance (PERMANOVA) via the adonis function of the “vegan” package (version 2.5.7) [33]. Ordination was performed using the Bray–Curtis dissimilarity-based principal coordinate analysis (PCoA). Differentially abundant OTUs between both groups at different time points were identified with the help of the R package “DESeq2” (version 1.32.0), which uses tests based on the negative binomial distribution [34] (Table S3). To detect OTUs that are differentially abundant in fecal samples of horses after vaccination compared to the time before vaccination, the R package “edgeR” (version 3.34.1) [35] for paired samples was used (Tables S4 and S5). Raw *p*-values were adjusted using the method of Benjamini and Hochberg [36] to control a false discovery rate (FDR) of 5%. Additionally, a cutoff for the log2 fold change of +/−1 was set. A Venn diagram was generated using the package “rstatix”.

The data of blood cells are expressed as mean ± SEM and the number of subjects as *n*. The data were tested for normal distribution using the SAS Enterprise Guide 7.1 program (SAS Institute, Inc., Cary, NC, USA) with Shapiro–Wilk, Kolmogorov–Smirnov, Cramer–von Mises, and Anderson–Darling tests. If no normal distribution was present, the data were transformed to achieve a normal distribution. A comparison of all of the parameters between the time points in each group was performed with a paired *T*-test for the normally distributed data. For non-normally distributed data, a Wilcoxon test was used. Previously, the variables per group were tested for normal distribution. To check which effects influence the data, a two-way analysis of variance was applied after testing the normal distribution, as described above, or a Wilcoxon test in the case of non-normally distributed data. For the data on the feeding process per group, a normal distribution was assumed for descriptive purposes, and a one-way analysis of variance of repeated measurements was performed. For all statistical methods, a *p*-value < 0.05 indicates significance.

## 3. Results

### 3.1. Fecal Microbiota Diversity

**Figure 2:**
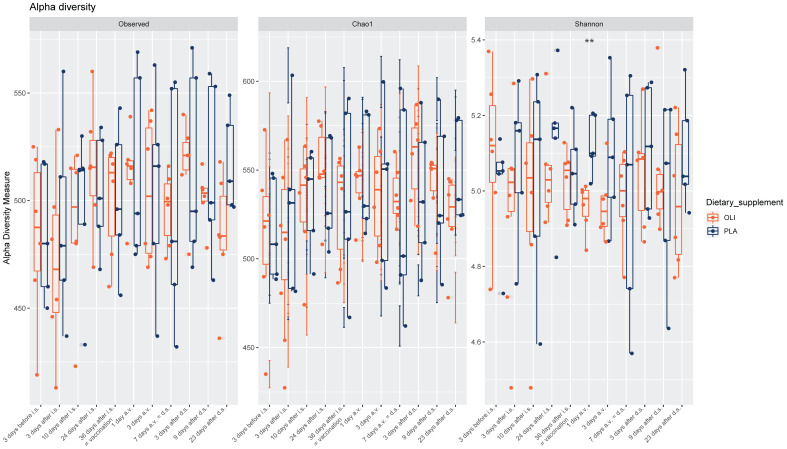
Box-plots showing Observed, Chao1 and Shannon index in fecal samples of horses depending on groups (OLI = Olimond, PLA = Placebo) separately for each sampling time (i.s. = introduction of the supplement, a.v. = after vaccination, d.s. = discontinuation of the supplement). **: *p*-value < 0.01.

No consistent picture was identified in terms of the possible effects of the dietary supplement on bacterial richness and diversity in fecal samples of horses (Figure 2). Shannon′s diversity index was statistically significantly different between the placebo and Olimond on the day after vaccination (Figure 2 and Table S2).

### 3.2. Heterogeneity of the Gut Microbial Community of the Individual Horse

Both dietary supplementation and sampled animals had a significant effect on the bacterial composition of the samples (Table 1).

**Figure 3:**
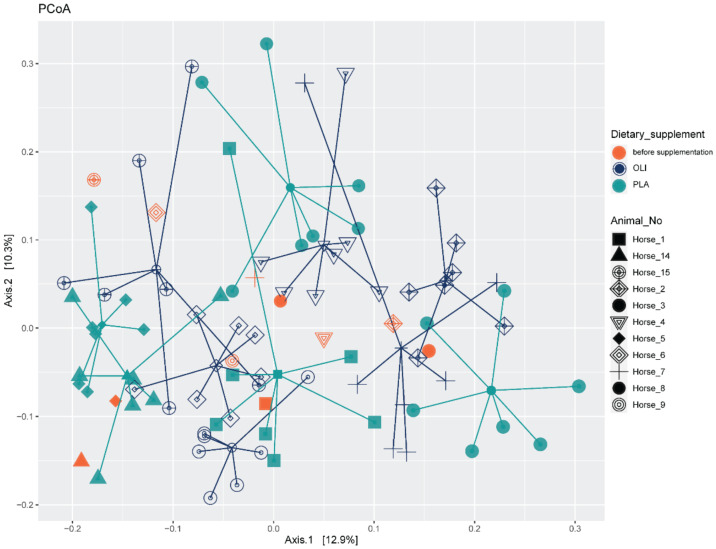
Bray–Curtis dissimilarity-based principal coordinate analysis (PcoA) was performed on fecal samples of horses. Different point shapes represent samples of a different horse; lines connect samples obtained from horses with the start of the experiment. Colors indicate the group membership (OLI = Olimond, PLA = Placebo). Samples marked in orange were taken before the start of the experiment.

Testing the prediction that microbiota would cluster after supplementation yielded a significant *p*-value; however, the R^2^ value was low (0.055), suggesting poor separation of the communities by this variable alone. Instead, the samples separated more clearly by the individual animal (*p* = 0.001, R^2^ = 0.484). The separation between microbiota composition of samples is visualized as PCoA in Figure 3.

### 3.3. Identification of Specific Taxa Associated with the Feeding Measures

Fecal microbiota was dominated at the phylum level by *Bacteroidetes* (average: 41.7%) and *Firmicutes* (33.3%), followed by *Verrucomicrobia* (12.6%), *Spirochaetae* (average: 6.20%), and *Fibrobacteres* (average: 4.11%). The relative abundances of bacterial phyla in every sample are shown in Figure S1.

The number of differentially abundant OTUs in the pairwise comparisons of the two groups are shown in Table 2. The differentially abundant OTUs between both groups at different time points are listed in Table S3.

**Figure 4:**
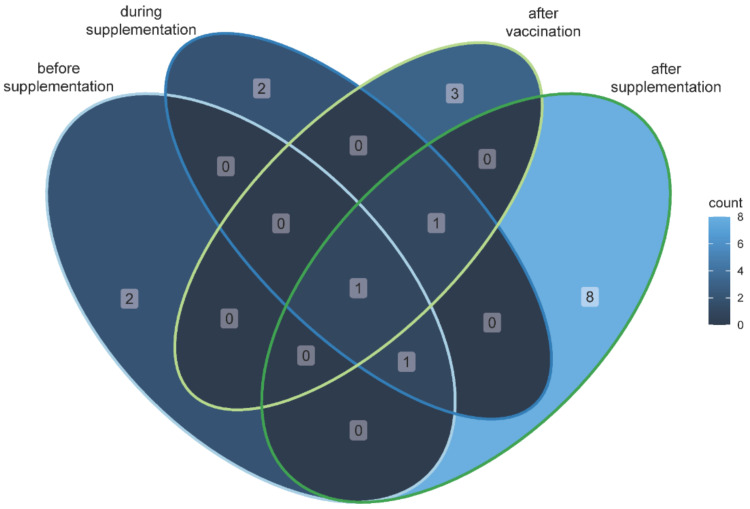
Venn diagram showing significantly different OTUs (selected with a criterion of FDR-adjusted *p*-values < 0.05 and absolute log2 fold change > 1) between horses belonging to the two groups PLA and OLI that are shared among the experimental phases.

A Venn diagram showing significantly different OTUs between OLI and PLA horses that are shared from day −3 to 0 (before supplementation), day 1–36 (six horses received the SCF product during supplementation), day 37–43 (the SCF product is still offered, but all horses were vaccinated at day 36, after vaccination), and day 43–66 (the horses received no supplementation, after supplementation) (Figure 4). Significantly different OTUs in the intersections of the Venn diagram seem to be influenced by the animals themselves (therefore appearing as significantly different in more than one experimental phase) rather than by administering the supplement.

OTU_54 was repeatedly identified in all experimental phases as significantly different between the PLA and OLI horses. OTU_54 was enriched in the samples from the PLA horses already before supplementation, the 10th and 24th day of offer, seven days after vaccination, and finally with the third day until the ninth day after the discontinuation of the supplementation (Table S3). OTU_54 represents a bacterial species that is assigned to the genus *Fibrobacter*, which belongs to the family *Fibrobacteraceae* within the phylum *Fibrobacteres*. Two OTUs (OTU_1205 and OTU_2034) were found to be significantly different between both groups exclusively during the offer of the supplements. Both OTUs were enriched in samples of PLA horses only once, 24 days after the introduction of the supplement. None of the OTUs identified as significantly different in abundance between the feeding groups up to the day of vaccination significantly changed its abundance in response to vaccination (Tables S4 and S5).

Three OTUs were identified as being significantly different between both groups exclusively three and seven days after vaccination: OTU_346 (phylum: *Spirochaetae*, family: *Spirochaetaceae*, genus *Treponema* 2), OTU_28 (phylum: *Bacteroidetes*, family: *Porphyromonadaceae*), and OTU_258 (phylum: *Verruccomicrobia*). Counts of OTU_28 in OLI horses showed a significant enrichment 3 days after vaccination. On the same day, this out was additionally identified as being significantly different between PLA and OLI horses, with higher read counts in PLA horses.

### 3.4. SCFP Feeding Does Not Alter Time-Dependend Changes of Blood Leukocyte Subpopulations

**Figure 5:**
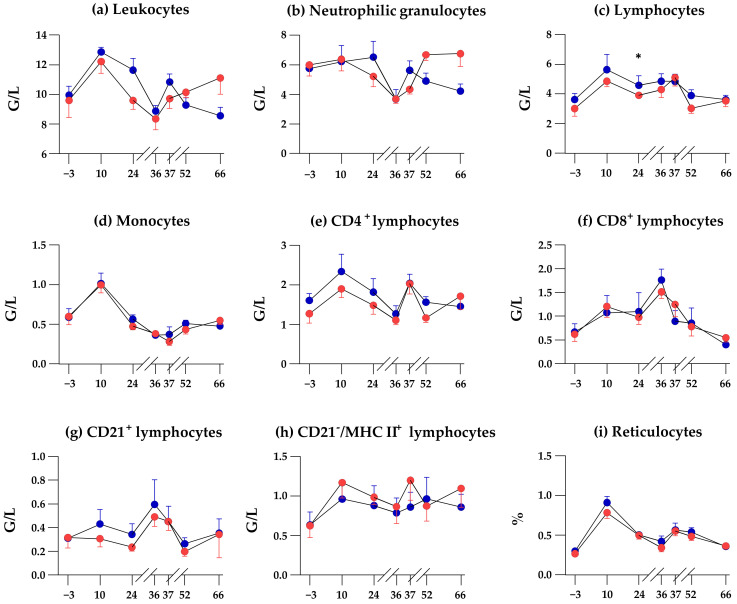
Changes in leukocyte counts ((**a**), blood leukocytes subpopulations; (**b**), neutrophilic granulocytes; (**c**), lymphocytes; (**d**), monocytes; (**e**), CD4^+^ T cells; (**f**), CD8^+^ T cells; (**g**), CD21^+^ B cells; (**h**) CD21 MHC II^+^ lymphocytes) and reticulocyte percentages (**i**) in the blood due to feeding. Cell counts of (**a**) and percentage of reticulocytes were determined by flow cytometry on days −3 (before supplementation), 10, 24 (during supplementation), 36 (during supplementation and day of vaccination), 37 (after vaccination), 52 and 66 (after supplementation). Shown are the responses per group (OLI in red, *n* = 6; PLA in blue, *n* = 5). The *p*-value was determined with the two-sample-test or the Wilcoxon test and describe the significance between the two groups per sampling day. Significance is present if the *p*-value is < 0.05.

The number of leukocytes, neutrophilic granulocytes, monocytes, CD4^+^/CD8^+^ T cells, CD21^+^ B cells, CD21^-^ MHCII^+^ cells, and the fraction of reticulocytes did not differ between the groups on sampling days −3 to 36 (with the exception on sampling day 28 for lymphocyte numbers, *p* = 0.036). Between sampling days, day 36 (day of vaccination) and day 66, time-dependent changes in leukocyte subpopulation numbers were the same in both groups, except for the apparent disparate kinetics of leucocytes and neutrophilic granulocyte numbers (Figure 5a,b). Factor time had the strongest effect on all cell populations (except CD21^−^/MHCII^+^ lymphocytes, Table 3). The interaction of group and time had a significant effect on leukocyte and neutrophilic granulocyte numbers. The group had no influence on any of the determined parameters (Table 3).

**Figure 6:**
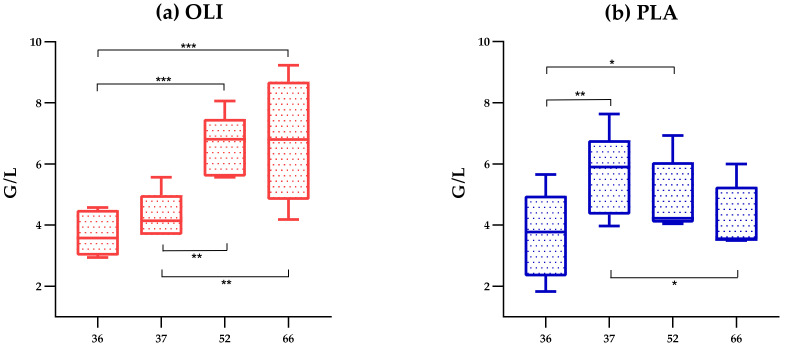
Changes in neutrophilic granulocytes per group ((**a**), OLI, *n* = 6; (**b**), PLA, *n* = 5) from day 36 to day 66 in the blood after vaccination. Cell counts were determined by flow cytometry on days 36 (during supplementation and before vaccination), 37 (during supplementation and after vaccination), 56, and 70 (after supplementation). One-way analysis of variance for repeated measurements (assumed normal distribution). * (*p* < 0.05), ** (*p <* 0.02), *** (*p <* 0.01). There is no significance between the two groups at any time point shown.

On the day of vaccination and at any time point after vaccination, OLI and PLA horses did not differ significantly in the number of neutrophilic granulocytes. However, changes in the numbers of neutrophilic granulocytes after vaccination appeared to be different between the PLA and OLI horses. In the OLI group, the numbers significantly rose between day 36 and day 66, whereas in the PLA group, the numbers significantly dropped from day 37 to day 66 after a significant rise between day 36 and day 37 (Figure 6).

## 4. Discussion

Diet has significant effects on the fecal microbiota of healthy horses [37]. We first addressed the question of whether SCFP feeding leads to an altered composition of fecal microbiota.

The trial was designed to allow for representative sampling. We investigated both short-term and long-term effects by taking samples before the start of supplementation and six samples during the 43-day feeding period (Figure 1). A similar duration of the feeding phase has been used in other studies on fecal microbiota [11,37,38]. The number of horses (*n* = 11) was in the range of other studies (between six and seventeen [11,37,38,39]). Our design largely excluded random influences on the microbiota that can occur on each sample day despite identical experimental conditions [40]. Changes in the bacterial community due to dietary components can be detected in the fecal microbiota after only a few days [12,40]. A dietary change in foals in response to pasture grass was visible in the fecal microbiota within 4 days [41].

No significant, consistent effect on fecal microbiota could be attributed to SCFP feeding. Despite the subtle differences in distinct OTUs, OLI, and PLA the animals did not differ in their alpha diversity during the feeding period up to the day of inoculation (day 36, Figure 2 and Table S2). Testing the prediction that microbiota would cluster after supplementation yielded a significant *p*-value; however, only 5.5% of the sample variability can be explained by this factor alone. This is in contrast to other studies that have found alterations in the composition of the gut microbiome due to the supplementation of feed with *Saccharomyces cerevisiae* fermentation products (cows [5], calves [6,7], dogs [8], and cats [9]). The largely unchanged composition of the microbiota after SCFP feeding compared with the placebo group was reflected to some extent in the unchanged time-dependent kinetics of the number and composition of blood leukocytes, their subpopulations, and the fraction of reticulocytes. During feeding until inoculation on day 36, the parameters of both groups showed an approximately parallel course, and, with one exception on day 28, there was no significant difference between the groups (Figure 5). The fact that SCFP feeding had no effect on circulating cells in the blood was also discussed by Lucassen et al. [19]. At this point, however, it must be emphasized that only the microbiota in the feces was analyzed to illustrate the potential influence of the supplement on the gut microbiota of the horses. The intestinal compartments (stomach, colon, small intestine, rectum) harbor bacterial species in different variations and abundances [42,43]. Therefore, in the absence of a significant shift in fecal bacterial species diversity by SCFP feeding, we cannot exclude the possibility that putative prebiotic effects manifested in the small intestine, the proximal colon, or in between.

The individual animal had the greatest influence on the composition of the fecal microbiota (Table 2). The horses in our study formed a homogeneous group kept under identical conditions. This precludes that husbandry conditions, training intensity, environmental factors, age, breed, and weather conditions had an influence on the highly dynamic microbiome [12,44,45]. Whether individual horses responded differently to physical and psychological stressors, as described in Mach et al., was not considered in our study [46].

An isolated OTU of the genus *Fibrobacter*, which belongs to the family *Fibrobacteraceae* (phylum *Fibrobacteres*), differed significantly between PLA and OLI horses. This OTU repeatedly and independent of the SCFP supplementation (day −3, day 10, day 24, day 43, day 46, and day 52) showed increased abundance in the PLA group (Table S3). *Fibrobacter* ferment a narrow range of carbohydrates, including cellulose and cellobiose, with the major fermentation products succinic and acetic acids, sometimes with a small amount of formic acid [47]. *Fibrobacteres* are major degraders of plant biomass in the herbivore gut [48]. The highest relative abundances of *Fibrobacteres* sequences are observed in samples from strictly herbivorous hosts with large body weights [49]. In horses, *Fibrobacter* is most abundant in the small intestine, large intestine, and rectum [44]. Previously, a significant increase in the relative abundance of *Fibrobacter* has been associated with the increased feeding of hay in horses [37,50] or in grass-fed compared to concentrate-fed horses [10]. Live yeast (*Saccharomyces cerevisiae*) supplementation in combination with a high-starch diet resulted in a significant reduction in relative levels of *Fibrobacter succinogenes* in fecal samples of horses, which was chosen as one of two exemplary cellulolytic bacteria [51]. *Fibrobacter* require carbon dioxide, straight-chain and branched-chain saturated fatty acids, and one or more vitamins, while ammonia is essential as the nitrogen source [47]. The major fermentation products are succinic and acetic acids, sometimes with a small amount of formic acid [47]. Two studies have observed lower abundances of the genus *Fibrobacter* in association with equine disease. In contrast to horses with Equine metabolic syndrome (EMS), an overrepresentation of the genus *Fibrobacter* is found in healthy control horses [52]. In the study by Leclere et al., healthy horses and horses with asthma were relocated to the same stables after a grazing period, while the change of feed to hay and of the stable environment resulted in a significant increase in the relative abundance of *Fibrobacter* only in healthy horses [37]. This indicates that the intestinal microbiota of asthmatic horses does not adapt in the same way to dietary and environmental changes as the microbiota of healthy horses. Underlying mechanisms that explain how airway obstruction and inflammation could influence the intestinal microbiota and how, in turn, this microbiota could modulate systemic inflammation in asthmatic horses deserves further investigation [37]. It should be emphasized that a significant horse effect was found for *Fibrobacteres* in the same study [37].

Interestingly, a parenteral vaccination against influenza resulted in a significant difference in alpha diversity between PLA and OLI horses (Figure 2). Alpha diversity increased in PLA horses, whereas it remained at the same level in OLI horses (Figure 2). Such a vaccine-induced change in the composition of the gut microbiome was described in cows [25] and in calves. A second vaccination with *E. coli* O157:H7 resulted in significantly different alpha diversity of the fecal microbiota [25]. Of the three measured diversity indices, only the Shannon index differed significantly between both groups. The Shannon index additionally takes the evenness of species in a community into account. While the total number of bacterial species has not changed (indicated by measured richness between PLA and OLI horses), there is a higher similar abundance between the present bacterial species within fecal microbiota of PLA horses compared to OLI horses one day after vaccination. We suspect that due to vaccination, no more bacterial species appear, but the abundance of the present species within microbiota was influenced differently between PLA and OLI horses.

The reasons for a challenge-induced change in the fecal microbiota are largely unknown but may be induced by circulating vaccine-induced cytokines and other mediators reaching the gut microbiome [53]. That SCFP feeding of racehorses may have altered the spectrum of released mediators after vaccination has been discussed previously [19]. In support of this, PLA and OLI horses showed a different trend in numbers of neutrophilic granulocytes, monocytes, CD4^+^ lymphocytes, CD8^+^ lymphocytes, and CD21^−^/MHCII^+^ lymphocytes between day 36 and day 66 (Figure 6 and Figure S2). That SCFP feeding alters a vaccination-induced mediator spectrum has also been reported for cattle [54] and poultry [55]. Local gut epithelial cells sensing vaccination-induced mediators may, in turn, release a different spectrum of mediators affecting the local microbiota [56,57,58]. Thus, the difference in alpha diversity between OLI and PLA horses 24 h after parenteral vaccination may be the consequence of a temporarily altered host-microbiota interaction. That such effects on the fecal microbiota could be seen within 24 h after the application of the parenteral stimulus was rather surprising. However, rapid microbiota changes in the feces of racehorses have been described after exposure to a race stressor in a time window between 24 h before and 48 h after the race [45].

## 5. Conclusions

Since feeding with the SCFP Olimond BB did not cause a significant shift in fecal bacterial species, a prebiotic effect of Olimond BB seems very unlikely. Differences between SCFP- and placebo-fed racehorses regarding a vaccination-induced altered alpha diversity of fecal microbiota and the development of blood leukocyte subset numbers support the hypothesis that postbiotic ingredients of Olimond BB altered the host response after vaccination. In sum, the findings indicate an immunomodulatory effect of SCFP-feeding, which is independent of significant microbiota changes in the gastrointestinal tract. The lack of a vaccination-induced change in the alpha diversity of SCFP-fed horses points towards SCPF-mediated regulatory mechanisms of inflammatory responses, which should be analyzed in the future.

## Figures and Tables

**Table 1:** Permutational multivariate analysis of variance (PERMANOVA) results based on Bray-–Curtis dissimilarities.

	DF	SumsOfSqs	F.Model	R^2^	Pr (>F)
Dietary_supplement	1	0.485	7.097	0.055	0.001
Animal_No	9	4.296	6.980	0.484	0.001
Residuals	60	4.103			
Total	70	8.884			

**Table 2:** Number of differentially abundant features in the pairwise comparisons of the two groups at different time points during the experiment.

Samples	Comparison	*p* < 0.05	pFDR < 0.05 & abs (log2FC) > 1
Day −3	PLA vs. OLI	53	4
Day 3	PLA vs. OLI	39	0
Day 10	PLA vs. OLI	35	1
Day 24	PLA vs. OLI	31	3
Day 36 ^1^	PLA vs. OLI	37	2
Day 37 ^2^	PLA vs. OLI	49	0
Day 39 ^3^	PLA vs. OLI	47	3
Day 43 ^4^	PLA vs. OLI	54	2
Day 46 ^5^	PLA vs. OLI	43	8
Day 52 ^6^	PLA vs. OLI	30	0
Day 66 ^7^	PLA vs. OLI	49	1

^1^ day of vaccination; ^2^ 24 h after vaccination; ^3^ 3 days after vaccination ^4^ 7 days after vaccination/ discontinuation of the supplement; ^5^ 3 days after discontinuation of the supplement; ^6^ 9 days after discontinuation of the supplement; ^7^ 23 days after discontinuation of the supplement. Raw *p*-values were adjusted using the method of Benjamini and Hochberg [36] to control a false discovery rate (FDR) of 5%. Additionally, a cutoff for the log2 fold change of +/−1 was set. PLA = Placebo, OLI = Olimond.

**Table 3:** Two-way analysis of variance (ANOVA).

	Point in Time	Group	Group * Point in Time ^1^
	Pr (>F)	Pr (>F)	Pr (>F)
Leukocytes	<0.0001	-	0.002
Neutrophilic granulocytes	<0.0001	-	0.002
Lymphocytes	<0.0001	-	-
Monocytes	<0.0001	-	-
CD4^+^ lymphocytes	<0.0001	-	-
CD8^+^ lymphocytes	<0.0001	-	-
CD21^+^ lymphocytes	0.006	-	-
CD21^−^/MHC II^+^ lymphocytes	-	-	-
Reticulocytes	<0.0001	-	-

The data were tested for normal distribution. If normal distribution was present, a two-way analysis of variance was performed. If not normally distributed, a Wilcoxon test was applied. Significance is present if the *p*-value is < 0.05. ^1,^*: Interaction between group and point in time.

## Data Availability

The data for this study have been deposited in the European Nucleotide Archive (ENA) at EMBL-EBI under accession number PRJEB52027.

## Supplementary Materials

The following supporting information can be downloaded at: https://www.mdpi.com/article/10.3390/ani12121496/s1, Figure S1: Bar charts represent the relative abundances of the dominant phyla in fecal samples of horses; Figure S2: Time effect of leukocyte subpopulations between day 36 and 66 per group; Table S1: Placebo and Olimond BB feed supplement ingredients; Table S2: Assessment of the treatment on alpha diversity estimates; Table S3: Differentially abundant OTUs (padj < 0.05) in samples of horses that have received Placebo vs Olimond at different time points of the experiment; Table S4: Differentially abundant OTUs (padj < 0.05) between two time points of horses that have received Olimond; Table S5. Differentially abundant OTUs (padj < 0.05) between two time points of horses that have received Placebo.
