# Supplementary material for: Feeding a Saccharomyces cerevisiae Fermentation Product (Olimond BB) Does Not Alter the Fecal Microbiota of Thoroughbred Racehorses
Source: Animals (Basel). 2022 Jun 8;12(12):1496. doi: 10.3390/ani12121496 (PMC9219515; doi:10.3390/ani12121496)
Supplement: Supplementary file 1 [file animals-12-01496-s001.zip › Figure S1.pdf]

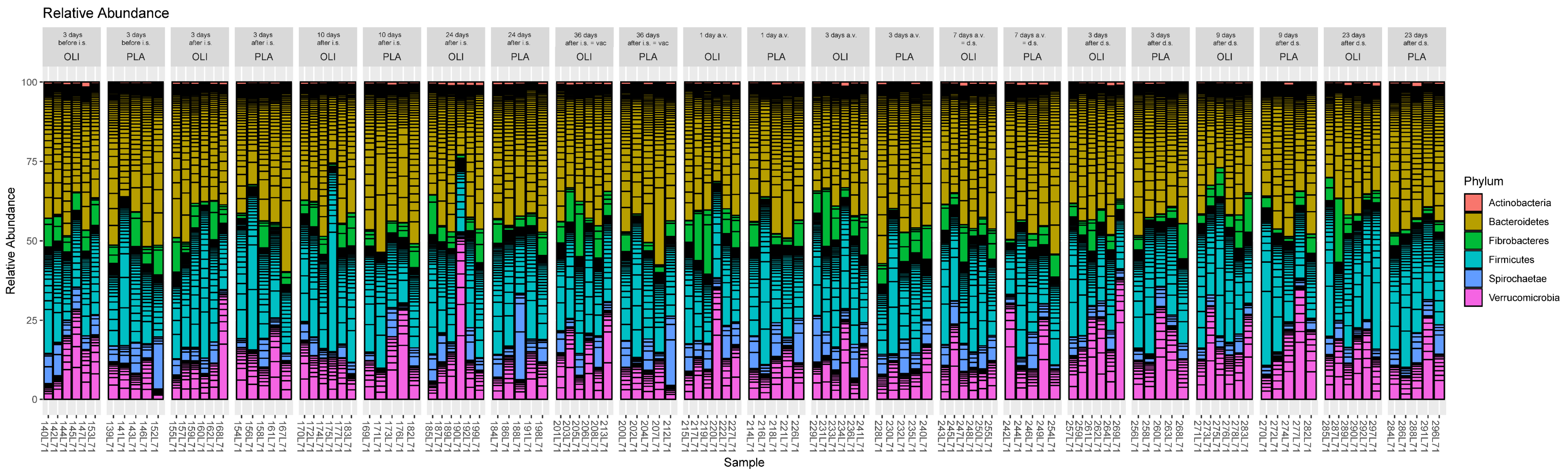

Figure S1. Bar charts represent the relative abundances of the dominant phyla in fecal samples of horses (i.s. = introduction of the supplement, a.v. = after vaccination, d.s. = discontinuation of the supplement).
