# Supplementary material for: Feeding a Saccharomyces cerevisiae Fermentation Product (Olimond BB) Does Not Alter the Fecal Microbiota of Thoroughbred Racehorses
Source: Animals (Basel). 2022 Jun 8;12(12):1496. doi: 10.3390/ani12121496 (PMC9219515; doi:10.3390/ani12121496)
Supplement: Supplementary file 1 [file animals-12-01496-s001.zip › Figure S2.pdf]

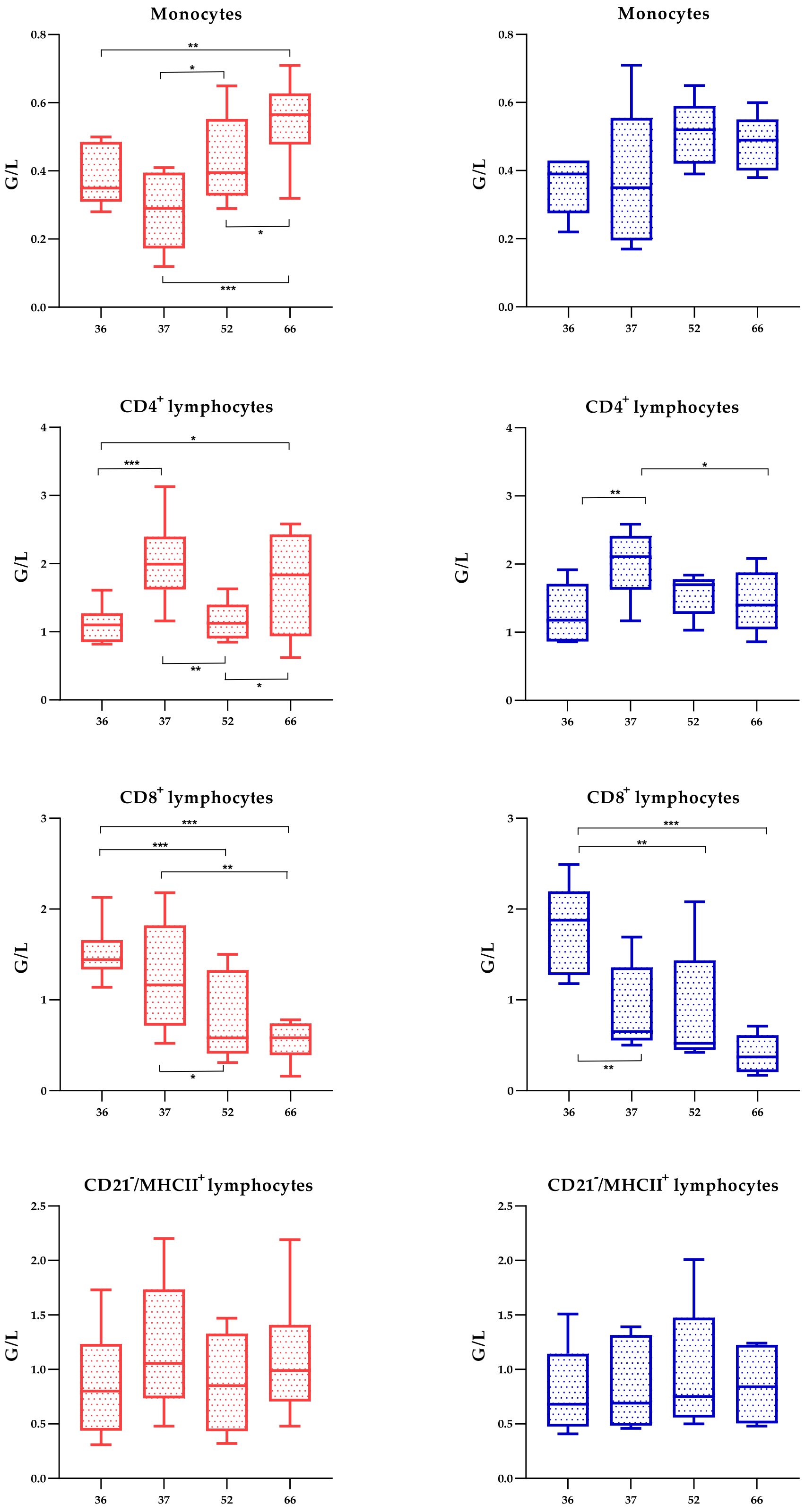

Figure S2: Time effect of leukocyte subpopulations between day 36 and 66 per group.Changes in leukocytes subpopulations per group (red bars: OLI, n = 6; blue bars: PLA, n = 5) from day 36 to day 66 in the blood after vaccination. Flow cytometry on days 36 (during supplementation and before vaccination), 37 (during supplementation and after vaccination), 56 and 70 determined cell counts (after supplementation). One-way analysis of variance for repeated measurements (assumed normal distribution). * ($p < 0.05$). There is no significance between the two groups at any time point shown.
