# Supplementary material for: Feeding a Saccharomyces cerevisiae Fermentation Product (Olimond BB) Does Not Alter the Fecal Microbiota of Thoroughbred Racehorses
Source: Animals (Basel). 2022 Jun 8;12(12):1496. doi: 10.3390/ani12121496 (PMC9219515; doi:10.3390/ani12121496)
Supplement: Supplementary file 1 [file animals-12-01496-s001.zip › Table S2.pdf]

Table S2: Assessment of the treatment on alpha diversity estimates.

Metrics	Mean ± SD Values		ANOVA Table (type II tests)		
	PLA	OLI	F	*p*-value	ges
			Richness		
Observed					
3 days before i. s.	485 ± 31.6	484 ± 39.3	0.005	0.947	0.0005
3 days after i. s.	490 ± 47.4	471 ± 42.2	0.503	0.496	0.053
10 days after i. s.	496 ± 38.3	489 ± 36.8		0.522[1]	
24 days after i. s.	504 ± 27.6	515 ± 30.7	0.397	0.544	0.042
36 days after i. s.	501 ± 34.4	501 ± 26.5	0.0003	0.986	3.68e-05
1 day a. v.	515 ± 44.8	513 ± 19.2	0.008	0.993	0.001
3 days a. v.	504 ± 47.9	504 ± 33.6	7.37e-06	0.998	8.19e-07
7 days a. v. = d. s.	496 ± 55.1	496 ± 17.0	2.00e-06	0.999	2.23e-07
3 days after d. s.	515 ± 46.3	516 ± 22.3	0.007	0.937	0.00074
9 days after d. s.	513 ± 41.5	501 ± 12.9	0.445	0.522	0.047
23 days after d. s.	518 ± 23.3	484 ± 28.7	4.406	0.065	0.329
Chao1					
3 days before i. s.	516 ± 28.8	513. ± 46.9	0.017	0.898	0.002
3 days after i. s.	528 ± 49.9	513 ± 41.4	0.294	0.601	0.032
10 days after i. s.	534 ± 29.5	531 ± 32.4	0.018	0.895	0.002
24 days after i. s.	537 ± 30.1	550 ± 25.2	0.650	0.441	0.067
36 days after i. s.	535 ± 51.4	530 ± 31.1	0.054	0.822	0.006
1 day a. v.	546 ± 33.3	542 ± 17.9	0.077	0.788	0.008
3 days a. v.	537 ± 46.6	536 ± 29.9	0.002	0.966	0.0002
7 days a. v. = d. s.	527 ± 59.5	536 ± 16.2	0.125	0.732	0.014
3 days after d. s.	537 ± 40.7	557 ± 26.2	0.993	0.345	0.099
9 days after d. s.	538 ± 41.6	541 ± 20.0	0.030	0.865	0.003
23 days after d. s.	548 ± 28.3	524 ± 25.0	2.313	0.163	0.204
			Diversity		
Shannon					
3 days before i. s.	5.01 ± 0.160	5.10 ± 0.219	0.609	0.455	0.063
3 days after i. s.	5.08 ± 0.209	5.01 ± 0.185	0.342	0.573	0.037
10 days after i. s.	5.03 ± 0.293	4.97 ± 0.284	0.105	0.754	0.012
24 days after i. s.	5.14 ± 0.197	5.05 ± 0.139	0.681	0.431	0.070
36 days after i. s.	5.05 ± 0.122	5.02 ± 0.089	0.173	0.687	0.019
1 day a. v.	5.12 ± 0.079	4.96 ± 0.065	14.80	**0.004**	0.622
3 days a. v.	5.10 ± 0.187	4.96 ± 0.093	2.398	0.156	0.210
7 days a. v. = d. s.	4.99 ± 0.321	4.98 ± 0.124	0.003	0.955	0.0004
3 days after d. s.	5.11 ± 0.170	5.05 ± 0.148	0.393	0.546	0.042
9 days after d. s.	5.00 ± 0.249	5.04 ± 0.173	0.115	0.742	0.013
23 days after d. s.	5.10 ± 0.151	4.98 ± 0.185	1.383	0.270	0.133

P-values < 0.05 are indicated in bold. 1 *P*-value was obtained using the non-parametric using Wilcoxon rank sum test. PLA = Placebo, OLI = Olimond, ges = generalized eta-squared, i.s. = introduction of the supplement, a. v. = after vaccination, d. s. = discontinuation of the supplement.
