# Supplementary material for: Feeding a Saccharomyces cerevisiae Fermentation Product (Olimond BB) Does Not Alter the Fecal Microbiota of Thoroughbred Racehorses
Source: Animals (Basel). 2022 Jun 8;12(12):1496. doi: 10.3390/ani12121496 (PMC9219515; doi:10.3390/ani12121496)
Supplement: Supplementary file 1 [file animals-12-01496-s001.zip › Table S4.pdf]

**Table S4**. Differentially abundant OTUs (padj < 0.05) between two time points of horses that have received Olimond.

| OTU | Phylum | Family | Genus | logFC | PValue | FDR |
|---|---|---|---|---|---|---|
| **Day of vs 1 day after vaccination** | | | | | | |
| 221 | Spirochaetae | *Spirochaetaceae* | *Treponema* 2 | -2.45 | 1.63e-05 | 0.0110 |
| **Before vs 3 days after vaccination** | | | | | | |
| 28 | Bacteroidetes | *Porphyromonadaceae* | *Ambiguous taxa* | -3.93 | 6.27e-06 | 0.0042 |
| **Before vs 7 days after vaccination** | | | | | | |
| 39 | Bacteroidetes | *Prevotellaceae* | *Alloprevotella* | -3.12 | 6.49e-05 | 0.0439 |
